# Bis[(*E*)-1-methyl-4-styrylpyridinium] 4-bromo­benzene­sulfonate iodide

**DOI:** 10.1107/S1600536810017277

**Published:** 2010-05-15

**Authors:** Chanasuk Surasit, Suchada Chantrapromma, Kullapa Chanawanno, Hoong-Kun Fun

**Affiliations:** a Crystal Materials Research Unit, Department of Chemistry, Faculty of Science, Prince of Songkla University, Hat-Yai, Songkhla 90112, Thailand; b X-ray Crystallography Unit, School of Physics, Universiti Sains Malaysia, 11800 USM, Penang, Malaysia

## Abstract

In the title compound, 2C_14_H_14_N^+^·C_6_H_4_BrO_3_S^−^·I^−^, two crystallographically independent cations exist in an *E* configuration with respect to the C=C ethenyl bond. One cation is approximately planar, whereas the other is twisted slightly, the dihedral angles between the pyridinium and phenyl rings of each cation being 0.96 (15) and 7.05 (16)°. In the crystal structure, the cations are stacked in an anti­parallel manner along the *a* axis through weak C—H⋯π inter­actions and π–π inter­actions, with centroid–centroid distances of 3.5544 (19) and 3.699 (2) Å. The 4-bromobenzene­sulfonate anions and the cations are linked together by weak C—H⋯O inter­actions. A short Br⋯I contact [3.6373 (4) Å] and C—H⋯I interactions are also observed.

## Related literature

For bond-length data, see: Allen *et al.* (1987 ▶). For background to non-linear optical materials research, see: Chia *et al.* (1995 ▶); Pan *et al.* (1996 ▶); Prasad & Williams (1991 ▶). For related structures, see: Chantrapromma *et al.* (2006 ▶); Fun, Chanawanno & Chantrapromma (2009*a* ▶,*b* ▶): Fun, Surasit *et al.* (2009 ▶). For the stability of the temperature controller used in the data collection, see: Cosier & Glazer (1986 ▶).
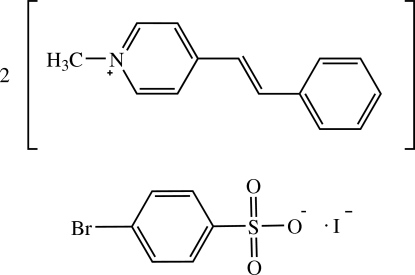


## Experimental

### 

#### Crystal data

2C_14_H_14_N^+^·C_6_H_4_BrO_3_S^−^·I^−^
*M* *_r_* = 755.49
Monoclinic, 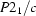

*a* = 7.7766 (2) Å
*b* = 32.2737 (9) Å
*c* = 12.8009 (4) Å
β = 96.097 (2)°
*V* = 3194.59 (16) Å^3^
*Z* = 4
Mo *K*α radiation
μ = 2.36 mm^−1^
*T* = 100 K
0.50 × 0.14 × 0.05 mm

#### Data collection

Bruker APEXII CCD area-detector diffractometer
Absorption correction: multi-scan (*SADABS*; Bruker, 2005 ▶) *T*_min_ = 0.383, *T*_max_ = 0.889
42790 measured reflections
9275 independent reflections
7161 reflections with *I* > 2σ(*I*)
*R*_int_ = 0.054

#### Refinement

*R*[*F* ^2^ > 2σ(*F* ^2^)] = 0.042
*wR*(*F* ^2^) = 0.089
*S* = 1.03
9275 reflections
381 parameters
H-atom parameters constrained
Δρ_max_ = 1.96 e Å^−3^
Δρ_min_ = −1.13 e Å^−3^

### 

Data collection: *APEX2* (Bruker, 2005 ▶); cell refinement: *SAINT* (Bruker, 2005 ▶); data reduction: *SAINT*; program(s) used to solve structure: *SHELXTL* (Sheldrick, 2008 ▶); program(s) used to refine structure: *SHELXTL*; molecular graphics: *SHELXTL*; software used to prepare material for publication: *SHELXTL* and *PLATON* (Spek, 2009 ▶).

## Supplementary Material

Crystal structure: contains datablocks global, I. DOI: 10.1107/S1600536810017277/is2540sup1.cif

Structure factors: contains datablocks I. DOI: 10.1107/S1600536810017277/is2540Isup2.hkl

Additional supplementary materials:  crystallographic information; 3D view; checkCIF report

## Figures and Tables

**Table 1:** Hydrogen-bond geometry (Å, °) *Cg*2 and *Cg*4 are the centroids of the C8*A*–C13*A* and C8*B*–C13*B* phenyl rings, respectively.

*D*—H⋯*A*	*D*—H	H⋯*A*	*D*⋯*A*	*D*—H⋯*A*
C2*A*—H2*AA*⋯O2^i^	0.93	2.45	3.253 (3)	144
C3*A*—H3*AA*⋯O1	0.93	2.47	3.189 (3)	134
C2*B*—H2*BA*⋯O2^ii^	0.93	2.24	3.169 (4)	177
C4*B*—H4*BA*⋯O3	0.93	2.49	3.328 (4)	151
C11*A*—H11*A*⋯O1^iii^	0.93	2.51	3.390 (4)	159
C7*B*—H7*BA*⋯O3	0.93	2.50	3.314 (4)	146
C14*A*—H14*C*⋯O1	0.96	2.44	3.171 (4)	133
C14*B*—H14*D*⋯O3^ii^	0.96	2.46	3.365 (4)	157
C1*A*—H1*AA*⋯I1^i^	0.93	3.26	3.841 (3)	123
C1*B*—H1*BA*⋯I1^ii^	0.93	3.35	3.787 (3)	111
C17—H17*A*⋯I1^iv^	0.93	3.10	3.863 (3)	141
C14*A*—H14*A*⋯*Cg*2^v^	0.96	2.72	3.475 (3)	136
C14*B*—H14*E*⋯*Cg*4^vi^	0.96	2.73	3.520 (3)	140

Symmetry codes: (i) 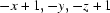; (ii) 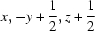; (iii) 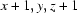; (iv) 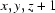; (v) 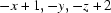; (vi) 
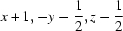
.

**Fig. 1.**
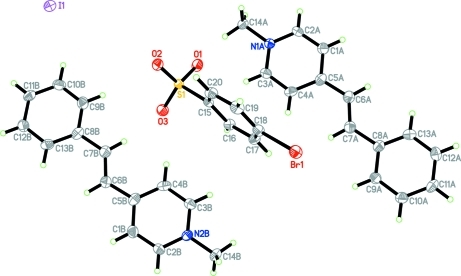
The molecular structure of the title compound, with 50% probability displacement ellipsoids and the atom-numbering scheme.

**Fig. 2.**
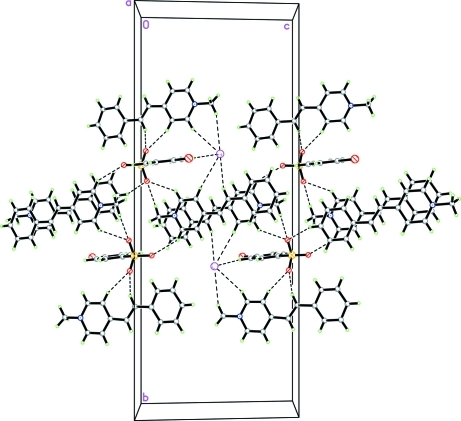
The crystal packing of the title compound viewed down the a axis. Weak C—H···O and C—H···I interactions are shown as dashed lines.

## supplementary crystallographic information

### Comment

Organic molecules are promising candidates for the nonlinear optical (NLO) applications. Stilbene derivatives, especially the pyridinium stilbenes with Donor-π-Acceptor system, were recognized as a good organic NLO chromophore (Chia *et al.*, 1995; Pan *et al.*, 1996). We previously reported the systhesis and crystal structure of bis[(*E*)-1-methyl-4-styrylpyridinium] 4-chlorobenzenesulfonate iodide (I), a pyridinium stilbene derivative, which crystallizes in noncentrosymmetric *P*2_1_ space group and exhibits second-order NLO properties (Fun *et al.*,  2009; Prasad & Williams, 1991). In this work, the title compound (II) was synthesized by changing the 4-chlorobenzenesulfonate anionic part in (I) to the 4-bromobenzenesulfonate to study the different NLO properties. By changing this, it was found that the title compound (II) crystallizes in centrosymmetric *P*2_1_/c space group and does not show second-order NLO properties.

The title molecule consists of two C_14_H_14_N^+^(*A* and *B*), one C_6_H_4_BrO_3_S^-^ and one I^-^ ions (Fig. 1), the two cations exist in an *E* configuration with respect to the C6═C7 ethenyl bond with the torsion angle of C6–C7–C8–C9 = 179.9 (3)° in molecule *A* [178.5 (3)° in molecule *B*]. One cation [molecule *A*] is planar while the other [molecule *B*] is slightly twisted, with the dihedral angles between the pyridinium and phenyl rings of the cation being 0.96 (15) and 7.05 (16)°, respectively. The two cations lie nearly on the same plane but in anti-parallel fashion with the dihedral angle between the planes through the whole molecule of cations being 4.01 (8)°. The anion is equally inclined with respect to the cations with the dihedral angles between the benzene ring of the anion and the pyridinium rings of the two cations being 82.20 (14) [molecule *A*] and 82.19 (15)° [molecule *B*], respectively. The bond distances in both cations and anion have normal values (Allen *et al.*, 1987) and comparable with the closely related compounds (Fun *et al.*, 2009*a*,*b*; Fun *et al.*,  2009).

In the crystal packing (Fig. 2), all O atoms of the sulfonate group are involved in weak C—H···O interactions (Table 1). The cations are stacked in an antiparallel manner along the *a* axis. The anions and I^-^ ions are located in interstitial spaces between the cations, and the ions linked together through weak C—H···O, C—H···I and C—H···π interactions (Table 1) forming a 3D network. The crystal structure is further stabilized by π–π interactions with the distances of Cg_1_···Cg_2_^vi^ = 3.5544 (19) Å and Cg_3_···Cg_4_^ii, vii^ = 3.699 (2) Å [(vi) = 2-x, -y, 2-z; (vii) = x, 1/2-y, =1/2+z; Cg_1_, Cg_2_, Cg_3_ and Cg_4_ are the centroids of C1A–C5A/N1A, C8A–C13A, C1B–C5B/N1B and C8B–C13B, respectively]. In addition the crystal structure also shows short C···O [3.169 (4)–3.365 (4) Å] and Br···I [3.6373 (4) Å] contacts.

### Experimental

(*E*)-1-methyl-4-styrylpyridinium iodide (compound A, 0.19 g, 0.58 mmol) which was prepared according the previous method (Fun *et al.*, 2009) was mixed with silver (I) 4-bromobenzenesulfonate (0.20 g, 0.58 mmol) (Chantrapromma *et al.*, 2006) in methanol solution and stirred for 30 minutes. The precipitate of silver iodide which formed was filtered and the filtrate was evaporated to give the title compound as an orange solid. Orange needle-shaped single crystals of the title compound suitable for *x*-ray structure determination were recrystallized from methanol by slow evaporation at room temperature over a week (m.p. 472–473 K).

### Refinement

All H atoms were positioned geometrically and allowed to ride on their parent atoms, with *d*(C—H) = 0.93 Å for aromatic and CH and 0.96 Å for CH_3_ atoms. The *U*_iso_(H) values were constrained to be 1.5*U*_eq_ of the carrier atom for methyl H atoms and 1.2*U*_eq_ for the remaining H atoms. A rotating group model was used for the methyl groups. The highest residual electron density peak is located at 0.78 Å from I1 and the deepest hole is located at 0.70 Å from I1.

### Crystal data

**Table:**

2C_14_H_14_N^+^·C_6_H_4_BrO_3_S^−^·I^−^	*F*(000) = 1512
*M**_r_* = 755.49	*D*_x_ = 1.571 Mg m^−^^3^
Monoclinic, *P*2_1_/*c*	Melting point = 472–473 K
Hall symbol: -P 2ybc	Mo *K*α radiation, λ = 0.71073 Å
*a* = 7.7766 (2) Å	Cell parameters from 9275 reflections
*b* = 32.2737 (9) Å	θ = 1.3–30.0°
*c* = 12.8009 (4) Å	µ = 2.36 mm^−^^1^
β = 96.097 (2)°	*T* = 100 K
*V* = 3194.59 (16) Å^3^	Needle, orange
*Z* = 4	0.50 × 0.14 × 0.05 mm

### Data collection

**Table:**

Bruker APEXII CCD area-detector diffractometer	9275 independent reflections
Radiation source: sealed tube	7161 reflections with *I* > 2σ(*I*)
graphite	*R*_int_ = 0.054
φ and ω scans	θ_max_ = 30.0°, θ_min_ = 1.3°
Absorption correction: multi-scan (*SADABS*; Bruker, 2005)	*h* = −10→10
*T*_min_ = 0.383, *T*_max_ = 0.889	*k* = −45→45
42790 measured reflections	*l* = −18→18

### Refinement

**Table:**

Refinement on *F*^2^	Primary atom site location: structure-invariant direct methods
Least-squares matrix: full	Secondary atom site location: difference Fourier map
*R*[*F*^2^ > 2σ(*F*^2^)] = 0.042	Hydrogen site location: inferred from neighbouring sites
*wR*(*F*^2^) = 0.089	H-atom parameters constrained
*S* = 1.03	*w* = 1/[σ^2^(*F*_o_^2^) + (0.0271*P*)^2^ + 6.2363*P*] where *P* = (*F*_o_^2^ + 2*F*_c_^2^)/3
9275 reflections	(Δ/σ)_max_ = 0.001
381 parameters	Δρ_max_ = 1.96 e Å^−^^3^
0 restraints	Δρ_min_ = −1.13 e Å^−^^3^

### Special details

**Table:**

Experimental. The crystal was placed in the cold stream of an Oxford Cryosystems Cobra open-flow nitrogen cryostat (Cosier & Glazer, 1986) operating at 100.0 (1) K.
Geometry. All esds (except the esd in the dihedral angle between two l.s. planes) are estimated using the full covariance matrix. The cell esds are taken into account individually in the estimation of esds in distances, angles and torsion angles; correlations between esds in cell parameters are only used when they are defined by crystal symmetry. An approximate (isotropic) treatment of cell esds is used for estimating esds involving l.s. planes.
Refinement. Refinement of F^2^ against ALL reflections. The weighted R-factor wR and goodness of fit S are based on F^2^, conventional R-factors R are based on F, with F set to zero for negative F^2^. The threshold expression of F^2^ > 2sigma(F^2^) is used only for calculating R-factors(gt) etc. and is not relevant to the choice of reflections for refinement. R-factors based on F^2^ are statistically about twice as large as those based on F, and R- factors based on ALL data will be even larger.

### Fractional atomic coordinates and isotropic or equivalent isotropic displacement parameters (Å^2^)

**Table:**

	*x*	*y*	*z*	*U*_iso_*/*U*_eq_	
I1	0.60308 (3)	0.140711 (6)	0.018059 (15)	0.02340 (6)	
Br1	1.22595 (4)	0.122562 (10)	0.83018 (3)	0.02557 (8)	
S1	0.51213 (10)	0.11278 (2)	0.52311 (5)	0.01778 (14)	
O1	0.4366 (3)	0.07387 (7)	0.55369 (17)	0.0263 (5)	
O2	0.5601 (3)	0.11252 (7)	0.41576 (16)	0.0247 (5)	
O3	0.4107 (3)	0.14897 (7)	0.54613 (17)	0.0244 (5)	
N1A	0.4474 (3)	−0.01295 (8)	0.72887 (19)	0.0188 (5)	
C1A	0.6113 (4)	−0.04541 (10)	0.8712 (2)	0.0247 (7)	
H1AA	0.6522	−0.0697	0.9044	0.030*	
C2A	0.5079 (4)	−0.04764 (9)	0.7787 (2)	0.0216 (6)	
H2AA	0.4787	−0.0734	0.7495	0.026*	
C3A	0.4852 (4)	0.02476 (10)	0.7710 (2)	0.0226 (6)	
H3AA	0.4414	0.0484	0.7363	0.027*	
C4A	0.5875 (4)	0.02843 (10)	0.8645 (2)	0.0241 (7)	
H4AA	0.6112	0.0545	0.8933	0.029*	
C5A	0.6568 (4)	−0.00702 (11)	0.9171 (2)	0.0244 (7)	
C6A	0.7692 (5)	−0.00622 (11)	1.0150 (3)	0.0282 (7)	
H6AA	0.8083	−0.0315	1.0433	0.034*	
C7A	0.8207 (4)	0.02818 (11)	1.0675 (2)	0.0268 (7)	
H7AA	0.7812	0.0533	1.0386	0.032*	
C8A	0.9350 (4)	0.02981 (10)	1.1676 (2)	0.0237 (6)	
C9A	0.9766 (4)	0.06887 (10)	1.2096 (3)	0.0265 (7)	
H9AA	0.9334	0.0925	1.1743	0.032*	
C10A	1.0815 (5)	0.07282 (11)	1.3031 (3)	0.0310 (8)	
H10A	1.1078	0.0991	1.3302	0.037*	
C11A	1.1479 (5)	0.03825 (11)	1.3571 (3)	0.0302 (8)	
H11A	1.2198	0.0412	1.4195	0.036*	
C12A	1.1059 (5)	−0.00100 (11)	1.3169 (3)	0.0296 (7)	
H12A	1.1479	−0.0245	1.3533	0.036*	
C13A	1.0021 (4)	−0.00511 (10)	1.2230 (2)	0.0250 (7)	
H13A	0.9764	−0.0314	1.1961	0.030*	
C14A	0.3408 (4)	−0.01642 (10)	0.6261 (2)	0.0235 (6)	
H14A	0.2455	−0.0348	0.6326	0.035*	
H14B	0.4103	−0.0271	0.5746	0.035*	
H14C	0.2974	0.0104	0.6045	0.035*	
N2B	0.5060 (3)	0.26563 (8)	0.85716 (19)	0.0202 (5)	
C1B	0.3607 (5)	0.30099 (11)	0.7133 (2)	0.0276 (7)	
H1BA	0.3248	0.3260	0.6821	0.033*	
C2B	0.4540 (4)	0.30119 (10)	0.8096 (2)	0.0235 (6)	
H2BA	0.4820	0.3263	0.8428	0.028*	
C3B	0.4697 (4)	0.22869 (10)	0.8097 (3)	0.0251 (7)	
H3BA	0.5070	0.2042	0.8433	0.030*	
C4B	0.3775 (5)	0.22730 (11)	0.7119 (3)	0.0280 (7)	
H4BA	0.3541	0.2019	0.6793	0.034*	
C5B	0.3181 (4)	0.26401 (11)	0.6607 (2)	0.0250 (7)	
C6B	0.2169 (5)	0.26553 (11)	0.5576 (3)	0.0284 (7)	
H6BA	0.1893	0.2914	0.5283	0.034*	
C7B	0.1624 (4)	0.23200 (11)	0.5035 (3)	0.0261 (7)	
H7BA	0.1938	0.2065	0.5334	0.031*	
C8B	0.0572 (4)	0.23131 (11)	0.4009 (2)	0.0247 (7)	
C9B	0.0042 (5)	0.19256 (11)	0.3613 (3)	0.0298 (7)	
H9BA	0.0390	0.1688	0.3989	0.036*	
C10B	−0.0981 (5)	0.18892 (11)	0.2678 (3)	0.0315 (8)	
H10B	−0.1333	0.1628	0.2431	0.038*	
C11B	−0.1495 (4)	0.22374 (11)	0.2099 (3)	0.0277 (7)	
H11B	−0.2180	0.2211	0.1461	0.033*	
C12B	−0.0986 (5)	0.26285 (11)	0.2471 (3)	0.0283 (7)	
H12B	−0.1333	0.2864	0.2085	0.034*	
C13B	0.0040 (4)	0.26660 (11)	0.3422 (3)	0.0273 (7)	
H13B	0.0378	0.2927	0.3672	0.033*	
C14B	0.6078 (4)	0.26654 (11)	0.9611 (2)	0.0266 (7)	
H14D	0.5600	0.2869	1.0046	0.040*	
H14E	0.7256	0.2736	0.9529	0.040*	
H14F	0.6041	0.2398	0.9934	0.040*	
C15	0.7120 (4)	0.11668 (9)	0.6057 (2)	0.0179 (6)	
C16	0.7080 (4)	0.12213 (9)	0.7132 (2)	0.0202 (6)	
H16A	0.6025	0.1247	0.7405	0.024*	
C17	0.8612 (4)	0.12368 (10)	0.7799 (2)	0.0225 (6)	
H17A	0.8593	0.1271	0.8519	0.027*	
C18	1.0171 (4)	0.12006 (9)	0.7367 (2)	0.0215 (6)	
C19	1.0250 (4)	0.11501 (10)	0.6301 (3)	0.0250 (7)	
H19A	1.1308	0.1128	0.6028	0.030*	
C20	0.8692 (4)	0.11333 (10)	0.5645 (2)	0.0236 (6)	
H20A	0.8712	0.1099	0.4925	0.028*	

### Atomic displacement parameters (Å^2^)

**Table:**

	*U*^11^	*U*^22^	*U*^33^	*U*^12^	*U*^13^	*U*^23^
I1	0.02846 (11)	0.02210 (10)	0.01938 (9)	0.00045 (9)	0.00141 (7)	−0.00226 (8)
Br1	0.01741 (15)	0.02441 (16)	0.03402 (17)	0.00048 (12)	−0.00127 (13)	−0.00088 (13)
S1	0.0203 (4)	0.0160 (3)	0.0170 (3)	−0.0016 (3)	0.0017 (3)	0.0009 (2)
O1	0.0293 (12)	0.0253 (12)	0.0232 (11)	−0.0098 (10)	−0.0021 (9)	0.0048 (9)
O2	0.0305 (13)	0.0245 (11)	0.0192 (10)	0.0025 (10)	0.0034 (9)	0.0011 (8)
O3	0.0225 (11)	0.0252 (12)	0.0257 (11)	0.0038 (9)	0.0034 (9)	−0.0014 (9)
N1A	0.0190 (13)	0.0197 (12)	0.0184 (11)	−0.0002 (10)	0.0050 (10)	−0.0004 (9)
C1A	0.0283 (17)	0.0236 (16)	0.0229 (15)	−0.0029 (13)	0.0063 (13)	0.0022 (12)
C2A	0.0225 (16)	0.0165 (14)	0.0267 (15)	0.0006 (12)	0.0073 (13)	−0.0007 (11)
C3A	0.0265 (17)	0.0196 (15)	0.0230 (14)	−0.0011 (12)	0.0082 (13)	0.0003 (11)
C4A	0.0281 (17)	0.0240 (16)	0.0213 (14)	−0.0066 (13)	0.0073 (13)	−0.0046 (12)
C5A	0.0232 (16)	0.0335 (18)	0.0173 (13)	−0.0032 (14)	0.0058 (12)	0.0010 (12)
C6A	0.0312 (19)	0.0277 (17)	0.0255 (15)	0.0004 (14)	0.0028 (14)	0.0011 (13)
C7A	0.0270 (17)	0.0295 (17)	0.0242 (15)	0.0037 (14)	0.0044 (13)	0.0007 (13)
C8A	0.0193 (15)	0.0313 (17)	0.0208 (14)	0.0019 (13)	0.0038 (12)	−0.0038 (12)
C9A	0.0256 (17)	0.0268 (16)	0.0268 (15)	0.0067 (14)	0.0015 (13)	−0.0033 (13)
C10A	0.0314 (19)	0.0281 (18)	0.0326 (17)	0.0040 (15)	−0.0009 (15)	−0.0076 (14)
C11A	0.0258 (17)	0.041 (2)	0.0224 (15)	−0.0006 (15)	−0.0038 (13)	−0.0034 (14)
C12A	0.0277 (18)	0.0294 (18)	0.0318 (17)	0.0023 (14)	0.0031 (15)	0.0080 (14)
C13A	0.0239 (16)	0.0255 (16)	0.0267 (15)	−0.0037 (13)	0.0072 (13)	−0.0045 (12)
C14A	0.0262 (17)	0.0244 (16)	0.0195 (14)	−0.0014 (13)	−0.0003 (12)	−0.0020 (11)
N2B	0.0190 (13)	0.0242 (13)	0.0176 (11)	−0.0011 (10)	0.0033 (10)	−0.0016 (10)
C1B	0.0317 (18)	0.0278 (17)	0.0237 (15)	0.0020 (14)	0.0050 (14)	0.0039 (13)
C2B	0.0249 (16)	0.0193 (15)	0.0270 (15)	−0.0008 (13)	0.0060 (13)	0.0002 (12)
C3B	0.0280 (17)	0.0206 (15)	0.0276 (16)	0.0001 (13)	0.0064 (13)	−0.0009 (12)
C4B	0.0305 (18)	0.0276 (17)	0.0266 (16)	−0.0058 (14)	0.0062 (14)	−0.0090 (13)
C5B	0.0212 (16)	0.0358 (18)	0.0192 (14)	−0.0014 (14)	0.0070 (12)	−0.0018 (12)
C6B	0.0339 (19)	0.0273 (17)	0.0237 (15)	0.0020 (14)	0.0017 (14)	0.0015 (13)
C7B	0.0240 (16)	0.0291 (17)	0.0255 (15)	0.0025 (14)	0.0033 (13)	−0.0006 (13)
C8B	0.0162 (15)	0.0366 (18)	0.0216 (14)	−0.0009 (13)	0.0033 (12)	−0.0024 (13)
C9B	0.0295 (18)	0.0304 (18)	0.0290 (16)	0.0077 (15)	0.0002 (14)	0.0013 (14)
C10B	0.0330 (19)	0.0252 (17)	0.0354 (18)	0.0026 (15)	−0.0007 (15)	−0.0064 (14)
C11B	0.0250 (17)	0.0336 (18)	0.0235 (15)	−0.0006 (14)	−0.0024 (13)	−0.0058 (13)
C12B	0.0286 (18)	0.0271 (17)	0.0287 (16)	−0.0003 (14)	0.0009 (14)	0.0030 (13)
C13B	0.0256 (17)	0.0286 (17)	0.0278 (16)	−0.0066 (14)	0.0041 (14)	−0.0057 (13)
C14B	0.0240 (17)	0.0354 (18)	0.0197 (14)	0.0006 (14)	−0.0002 (13)	−0.0017 (13)
C15	0.0182 (14)	0.0139 (13)	0.0217 (13)	−0.0001 (11)	0.0025 (11)	0.0005 (10)
C16	0.0153 (14)	0.0228 (15)	0.0229 (14)	−0.0004 (12)	0.0040 (11)	−0.0009 (11)
C17	0.0223 (15)	0.0237 (15)	0.0216 (14)	−0.0004 (13)	0.0028 (12)	−0.0012 (12)
C18	0.0169 (14)	0.0197 (15)	0.0275 (15)	−0.0005 (12)	0.0007 (12)	0.0001 (11)
C19	0.0174 (15)	0.0269 (16)	0.0321 (16)	−0.0002 (13)	0.0082 (13)	−0.0007 (13)
C20	0.0262 (17)	0.0231 (15)	0.0228 (14)	−0.0004 (13)	0.0088 (13)	−0.0011 (12)

### Geometric parameters (Å, °)

**Table:**

Br1—C18	1.914 (3)	C1B—C2B	1.363 (4)
S1—O3	1.457 (2)	C1B—C5B	1.392 (5)
S1—O1	1.457 (2)	C1B—H1BA	0.9300
S1—O2	1.462 (2)	C2B—H2BA	0.9300
S1—C15	1.789 (3)	C3B—C4B	1.376 (4)
N1A—C2A	1.348 (4)	C3B—H3BA	0.9300
N1A—C3A	1.351 (4)	C4B—C5B	1.407 (5)
N1A—C14A	1.483 (4)	C4B—H4BA	0.9300
C1A—C2A	1.361 (4)	C5B—C6B	1.465 (4)
C1A—C5A	1.400 (5)	C6B—C7B	1.330 (5)
C1A—H1AA	0.9300	C6B—H6BA	0.9300
C2A—H2AA	0.9300	C7B—C8B	1.472 (4)
C3A—C4A	1.370 (4)	C7B—H7BA	0.9300
C3A—H3AA	0.9300	C8B—C9B	1.395 (5)
C4A—C5A	1.406 (5)	C8B—C13B	1.402 (5)
C4A—H4AA	0.9300	C9B—C10B	1.370 (5)
C5A—C6A	1.449 (4)	C9B—H9BA	0.9300
C6A—C7A	1.337 (5)	C10B—C11B	1.382 (5)
C6A—H6AA	0.9300	C10B—H10B	0.9300
C7A—C8A	1.481 (4)	C11B—C12B	1.392 (5)
C7A—H7AA	0.9300	C11B—H11B	0.9300
C8A—C9A	1.395 (5)	C12B—C13B	1.388 (4)
C8A—C13A	1.402 (5)	C12B—H12B	0.9300
C9A—C10A	1.381 (4)	C13B—H13B	0.9300
C9A—H9AA	0.9300	C14B—H14D	0.9600
C10A—C11A	1.383 (5)	C14B—H14E	0.9600
C10A—H10A	0.9300	C14B—H14F	0.9600
C11A—C12A	1.393 (5)	C15—C20	1.386 (4)
C11A—H11A	0.9300	C15—C16	1.390 (4)
C12A—C13A	1.381 (5)	C16—C17	1.391 (4)
C12A—H12A	0.9300	C16—H16A	0.9300
C13A—H13A	0.9300	C17—C18	1.390 (4)
C14A—H14A	0.9600	C17—H17A	0.9300
C14A—H14B	0.9600	C18—C19	1.382 (4)
C14A—H14C	0.9600	C19—C20	1.401 (4)
N2B—C2B	1.341 (4)	C19—H19A	0.9300
N2B—C3B	1.354 (4)	C20—H20A	0.9300
N2B—C14B	1.474 (4)		
			
O3—S1—O1	113.20 (14)	N2B—C2B—C1B	120.8 (3)
O3—S1—O2	113.12 (13)	N2B—C2B—H2BA	119.6
O1—S1—O2	113.44 (13)	C1B—C2B—H2BA	119.6
O3—S1—C15	106.21 (13)	N2B—C3B—C4B	120.0 (3)
O1—S1—C15	104.57 (13)	N2B—C3B—H3BA	120.0
O2—S1—C15	105.29 (14)	C4B—C3B—H3BA	120.0
C2A—N1A—C3A	120.6 (3)	C3B—C4B—C5B	120.6 (3)
C2A—N1A—C14A	119.4 (3)	C3B—C4B—H4BA	119.7
C3A—N1A—C14A	120.0 (3)	C5B—C4B—H4BA	119.7
C2A—C1A—C5A	120.7 (3)	C1B—C5B—C4B	116.6 (3)
C2A—C1A—H1AA	119.6	C1B—C5B—C6B	119.0 (3)
C5A—C1A—H1AA	119.6	C4B—C5B—C6B	124.5 (3)
N1A—C2A—C1A	120.8 (3)	C7B—C6B—C5B	123.6 (3)
N1A—C2A—H2AA	119.6	C7B—C6B—H6BA	118.2
C1A—C2A—H2AA	119.6	C5B—C6B—H6BA	118.2
N1A—C3A—C4A	120.6 (3)	C6B—C7B—C8B	126.4 (3)
N1A—C3A—H3AA	119.7	C6B—C7B—H7BA	116.8
C4A—C3A—H3AA	119.7	C8B—C7B—H7BA	116.8
C3A—C4A—C5A	120.4 (3)	C9B—C8B—C13B	118.3 (3)
C3A—C4A—H4AA	119.8	C9B—C8B—C7B	116.9 (3)
C5A—C4A—H4AA	119.8	C13B—C8B—C7B	124.7 (3)
C1A—C5A—C4A	116.9 (3)	C10B—C9B—C8B	121.1 (3)
C1A—C5A—C6A	118.7 (3)	C10B—C9B—H9BA	119.5
C4A—C5A—C6A	124.4 (3)	C8B—C9B—H9BA	119.5
C7A—C6A—C5A	124.8 (3)	C9B—C10B—C11B	120.5 (3)
C7A—C6A—H6AA	117.6	C9B—C10B—H10B	119.8
C5A—C6A—H6AA	117.6	C11B—C10B—H10B	119.8
C6A—C7A—C8A	125.8 (3)	C10B—C11B—C12B	119.9 (3)
C6A—C7A—H7AA	117.1	C10B—C11B—H11B	120.1
C8A—C7A—H7AA	117.1	C12B—C11B—H11B	120.1
C9A—C8A—C13A	118.2 (3)	C13B—C12B—C11B	119.7 (3)
C9A—C8A—C7A	117.3 (3)	C13B—C12B—H12B	120.1
C13A—C8A—C7A	124.5 (3)	C11B—C12B—H12B	120.1
C10A—C9A—C8A	120.6 (3)	C12B—C13B—C8B	120.5 (3)
C10A—C9A—H9AA	119.7	C12B—C13B—H13B	119.7
C8A—C9A—H9AA	119.7	C8B—C13B—H13B	119.7
C9A—C10A—C11A	120.9 (3)	N2B—C14B—H14D	109.5
C9A—C10A—H10A	119.6	N2B—C14B—H14E	109.5
C11A—C10A—H10A	119.6	H14D—C14B—H14E	109.5
C10A—C11A—C12A	119.3 (3)	N2B—C14B—H14F	109.5
C10A—C11A—H11A	120.4	H14D—C14B—H14F	109.5
C12A—C11A—H11A	120.4	H14E—C14B—H14F	109.5
C13A—C12A—C11A	120.1 (3)	C20—C15—C16	120.0 (3)
C13A—C12A—H12A	120.0	C20—C15—S1	121.0 (2)
C11A—C12A—H12A	120.0	C16—C15—S1	119.0 (2)
C12A—C13A—C8A	121.0 (3)	C15—C16—C17	120.3 (3)
C12A—C13A—H13A	119.5	C15—C16—H16A	119.9
C8A—C13A—H13A	119.5	C17—C16—H16A	119.9
N1A—C14A—H14A	109.5	C18—C17—C16	118.6 (3)
N1A—C14A—H14B	109.5	C18—C17—H17A	120.7
H14A—C14A—H14B	109.5	C16—C17—H17A	120.7
N1A—C14A—H14C	109.5	C19—C18—C17	122.3 (3)
H14A—C14A—H14C	109.5	C19—C18—Br1	119.9 (2)
H14B—C14A—H14C	109.5	C17—C18—Br1	117.8 (2)
C2B—N2B—C3B	120.8 (3)	C18—C19—C20	118.1 (3)
C2B—N2B—C14B	120.0 (3)	C18—C19—H19A	121.0
C3B—N2B—C14B	119.3 (3)	C20—C19—H19A	121.0
C2B—C1B—C5B	121.2 (3)	C15—C20—C19	120.7 (3)
C2B—C1B—H1BA	119.4	C15—C20—H20A	119.7
C5B—C1B—H1BA	119.4	C19—C20—H20A	119.7
			
C3A—N1A—C2A—C1A	−1.4 (5)	C3B—C4B—C5B—C1B	1.2 (5)
C14A—N1A—C2A—C1A	177.6 (3)	C3B—C4B—C5B—C6B	−179.0 (3)
C5A—C1A—C2A—N1A	0.2 (5)	C1B—C5B—C6B—C7B	−176.5 (3)
C2A—N1A—C3A—C4A	0.8 (5)	C4B—C5B—C6B—C7B	3.7 (6)
C14A—N1A—C3A—C4A	−178.2 (3)	C5B—C6B—C7B—C8B	178.5 (3)
N1A—C3A—C4A—C5A	1.0 (5)	C6B—C7B—C8B—C9B	−175.7 (4)
C2A—C1A—C5A—C4A	1.5 (5)	C6B—C7B—C8B—C13B	2.7 (6)
C2A—C1A—C5A—C6A	−179.2 (3)	C13B—C8B—C9B—C10B	−0.4 (5)
C3A—C4A—C5A—C1A	−2.1 (5)	C7B—C8B—C9B—C10B	178.1 (3)
C3A—C4A—C5A—C6A	178.6 (3)	C8B—C9B—C10B—C11B	0.8 (6)
C1A—C5A—C6A—C7A	−179.1 (3)	C9B—C10B—C11B—C12B	−0.7 (6)
C4A—C5A—C6A—C7A	0.1 (6)	C10B—C11B—C12B—C13B	0.2 (5)
C5A—C6A—C7A—C8A	179.9 (3)	C11B—C12B—C13B—C8B	0.2 (5)
C6A—C7A—C8A—C9A	178.9 (4)	C9B—C8B—C13B—C12B	−0.1 (5)
C6A—C7A—C8A—C13A	−1.7 (6)	C7B—C8B—C13B—C12B	−178.5 (3)
C13A—C8A—C9A—C10A	0.1 (5)	O3—S1—C15—C20	−128.7 (3)
C7A—C8A—C9A—C10A	179.5 (3)	O1—S1—C15—C20	111.4 (3)
C8A—C9A—C10A—C11A	0.2 (6)	O2—S1—C15—C20	−8.4 (3)
C9A—C10A—C11A—C12A	−0.9 (6)	O3—S1—C15—C16	52.9 (3)
C10A—C11A—C12A—C13A	1.4 (5)	O1—S1—C15—C16	−67.0 (3)
C11A—C12A—C13A—C8A	−1.1 (5)	O2—S1—C15—C16	173.2 (2)
C9A—C8A—C13A—C12A	0.3 (5)	C20—C15—C16—C17	−0.9 (4)
C7A—C8A—C13A—C12A	−179.0 (3)	S1—C15—C16—C17	177.5 (2)
C3B—N2B—C2B—C1B	1.2 (5)	C15—C16—C17—C18	0.6 (5)
C14B—N2B—C2B—C1B	179.6 (3)	C16—C17—C18—C19	0.0 (5)
C5B—C1B—C2B—N2B	−0.7 (5)	C16—C17—C18—Br1	179.7 (2)
C2B—N2B—C3B—C4B	−0.4 (5)	C17—C18—C19—C20	−0.3 (5)
C14B—N2B—C3B—C4B	−178.8 (3)	Br1—C18—C19—C20	180.0 (2)
N2B—C3B—C4B—C5B	−0.9 (5)	C16—C15—C20—C19	0.6 (5)
C2B—C1B—C5B—C4B	−0.5 (5)	S1—C15—C20—C19	−177.8 (2)
C2B—C1B—C5B—C6B	179.7 (3)	C18—C19—C20—C15	0.0 (5)

### Hydrogen-bond geometry (Å, °)

**Table:**

Cg2 and Cg4 are the centroids of the C8A–C13A and C8B–C13B phenyl rings, respectively.

**Table:**

*D*—H···*A*	*D*—H	H···*A*	*D*···*A*	*D*—H···*A*
C2A—H2AA···O2^i^	0.93	2.45	3.253 (3)	144
C3A—H3AA···O1	0.93	2.47	3.189 (3)	134
C2B—H2BA···O2^ii^	0.93	2.24	3.169 (4)	177
C4B—H4BA···O3	0.93	2.49	3.328 (4)	151
C11A—H11A···O1^iii^	0.93	2.51	3.390 (4)	159
C7B—H7BA···O3	0.93	2.50	3.314 (4)	146
C14A—H14C···O1	0.96	2.44	3.171 (4)	133
C14B—H14D···O3^ii^	0.96	2.46	3.365 (4)	157
C1A—H1AA···I1^i^	0.93	3.26	3.841 (3)	123
C1B—H1BA···I1^ii^	0.93	3.35	3.787 (3)	111
C17—H17A···I1^iv^	0.93	3.10	3.863 (3)	141
C14A—H14A···Cg2^v^	0.96	2.72	3.475 (3)	136
C14B—H14E···Cg4^vi^	0.96	2.73	3.520 (3)	140

Symmetry codes: (i) −*x*+1, −*y*, −*z*+1;  (ii) *x*, −*y*+1/2, *z*+1/2; (iii) *x*+1, *y*, *z*+1; (iv) *x*, *y*, *z*+1; (v) −*x*+1, −*y*, −*z*+2; (vi) *x*+1, −*y*−1/2, *z*−1/2.
